# First implementation of dynamic oxygen-17 (^17^O) magnetic resonance imaging at 7 Tesla during neuronal stimulation in the human brain

**DOI:** 10.1007/s10334-023-01119-6

**Published:** 2023-09-22

**Authors:** Louise Ebersberger, Fabian J. Kratzer, Vanessa L. Franke, Armin M. Nagel, Sebastian C. Niesporek, Andreas Korzowski, Mark E. Ladd, Heinz-Peter Schlemmer, Daniel Paech, Tanja Platt

**Affiliations:** 1 German Cancer Research Center (DKFZ) Heidelberg, Division of Radiology, Heidelberg, Germany; 2 Faculty of Medicine, Ruprecht-Karls University Heidelberg, Heidelberg, Germany; 3 Department of Pediatrics, Bern University Hospital, Bern, Switzerland; 4 German Cancer Research Center (DKFZ) Heidelberg, Division of Medical Physics in Radiology, Heidelberg, Germany; 5 Faculty of Physics and Astronomy, Ruprecht-Karls University Heidelberg, Heidelberg, Germany; 6 Institute of Radiology, Friedrich-Alexander University Hospital Erlangen-Nürnberg (FAU), University Hospital Erlangen, Erlangen, Germany; 7 Department of Neuroradiology, University Hospital Bonn, Bonn, Germany

**Keywords:** Ultrahigh field MRI, Cerebral oxygen metabolism, Neuronal activity, Dynamic oxygen-17 imaging, fMRI

## Abstract

**Objective:**

First implementation of dynamic oxygen-17 (^17^O) MRI at 7 Tesla (T) during neuronal stimulation in the human brain.

**Methods:**

Five healthy volunteers underwent a three-phase ^17^O gas (^17^O_2_) inhalation experiment. Combined right-side visual stimulus and right-hand finger tapping were used to achieve neuronal stimulation in the left cerebral hemisphere. Data analysis included the evaluation of the relative partial volume (PV)-corrected time evolution of absolute ^17^O water (H_2_^17^O) concentration and of the relative signal evolution without PV correction. Statistical analysis was performed using a one-tailed paired *t* test. Blood oxygen level-dependent (BOLD) experiments were performed to validate the stimulation paradigm.

**Results:**

The BOLD maps showed significant activity in the stimulated left visual and sensorimotor cortex compared to the non-stimulated right side. PV correction of ^17^O MR data resulted in high signal fluctuations with a noise level of 10% due to small regions of interest (ROI), impeding further quantitative analysis. Statistical evaluation of the relative H_2_^17^O signal with PV correction (*p* = 0.168) and without (*p* = 0.382) did not show significant difference between the stimulated left and non-stimulated right sensorimotor ROI.

**Discussion:**

The change of cerebral oxygen metabolism induced by sensorimotor and visual stimulation is not large enough to be reliably detected with the current setup and methodology of dynamic ^17^O MRI at 7 T.

**Supplementary Information:**

The online version contains supplementary material available at 10.1007/s10334-023-01119-6.

## Introduction

The effect of neuronal activity on the brain’s energy consumption and the mapping of it are main areas of research in cognitive neuroscience [1]. Blood oxygen level-dependent (BOLD) imaging is an established method for non-invasively investigating neuronal activity with the advantage of not holding major obstacles for application [2]. This method, used in functional MRI (fMRI), provides an indirect measure for brain activation by correlating the changes in blood flow, blood volume and the oxygenation of hemoglobin with neuronal activity [3]. However, the signal changes are small, in the order of < 1% for many cognitive processes [1], and are susceptible to confounding factors, such as intrinsic noise and artifacts, which adds difficulty to data interpretation [4]. Secondly, BOLD imaging does not provide a direct measurement of neuronal oxygen consumption [5]. Instead, it is a compound metric and many factors influence the amount by which the BOLD response reflects the hemodynamic response, making quantification of this response challenging [6].

Dynamic ^17^O MRI on the other hand is a promising research tool that is able to provide direct metabolic information, e.g., after inhalation of the non-radioactive oxygen-17 isotope [7]. The inhaled oxygen-17 gas (^17^O_2_) remains undetectable until it is metabolized to oxygen-17 water (H_2_^17^O), thus ensuring that a signal increase after inhalation originates from metabolic activity. Atkinson and Thulborn described a metabolic model for determining the cerebral metabolic rate of oxygen consumption (CMRO_2_) in ^17^O measurements in the human brain. In their experiments, ^17^O-labeled water was investigated in an inhalation experiment of ^17^O-enriched oxygen gas to show the feasibility of determining CMRO_2_ in a healthy volunteer at 9.4 Tesla (T) [8]. A few years later, Hoffmann et al. designed and applied an efficient breathing system for ^17^O MRI at 7 T [9]. In 2014, the feasibility of cerebral and cardiac ^17^O MRI at 3 T was demonstrated on a healthy volunteer by Borowiak et al. [10]. The reproducibility of CMRO_2_ determination in a small cohort of healthy volunteers was shown by Niesporek et al. [11]. In clinical research, dynamic ^17^O MRI has mainly been applied in brain tumor imaging. The first clinical research exam employing dynamic ^17^O MRI was performed on a patient with glioblastoma at 7 T [12]. In this study the feasibility of assessing the CMRO_2_ in tumor tissue and the importance of partial volume (PV) correction for data analysis was demonstrated [12]. Following this, Paech et al. performed the first dynamic ^17^O MRI study including ten patients with newly diagnosed glioma. In accordance with the Warburg effect, a reduction of oxidative glycolysis could be shown for both, high- and low-grade glioma [13]. Recently, dynamic ^17^O MRI has been applied to a patient with early subacute stroke, not showing any significant difference between the stroke region and the mirrored healthy contralateral side, but motivating the application of this modality to patients with larger strokes [14].

^17^O MRI is a unique method that enables a direct and non-invasive assessment of cerebral oxygen metabolism and is thus interesting for studying physiological effects during neuronal activity. The effect of visual stimulation on the cerebral oxygen metabolism in cats has been investigated using ^17^O magnetic resonance spectroscopy (MRS), showing a significant increase of CMRO_2_ during stimulation compared to the resting state [15]. A few years later, Zhu et al. published an abstract with preliminary results examining the effect of visual stimulation on the human brain in one participant using ^17^O MRS, revealing a > 10% increase of CMRO_2_ in the occipital lobe [16]. However, ^17^O MRI for functional imaging has not yet been studied in a group of volunteers, so far.

The aim of this study was to evaluate the feasibility of detecting neuronal stimulation using dynamic ^17^O MRI at 7 T. For this purpose, dynamic oxygen data of five healthy volunteers undergoing combined sensorimotor and visual stimulation were acquired and compared to fMRI results.

## Materials and methods

### Study design and study participants

Five healthy right-handed volunteers (three females, age: 23, 23, 24, 29, 69) were included in this study. Written informed consent from all participants according to the institutional guidelines and approval from the local ethics committee were obtained prior to examinations. Study participants with refractive errors wore contact lenses throughout the measurement. A combination of visual and sensorimotor stimulation, explained below, was applied to yield a large, stimulated volume. BOLD images were used to validate the stimulation paradigm.

### Stimulation paradigm

**Fig. 1:**
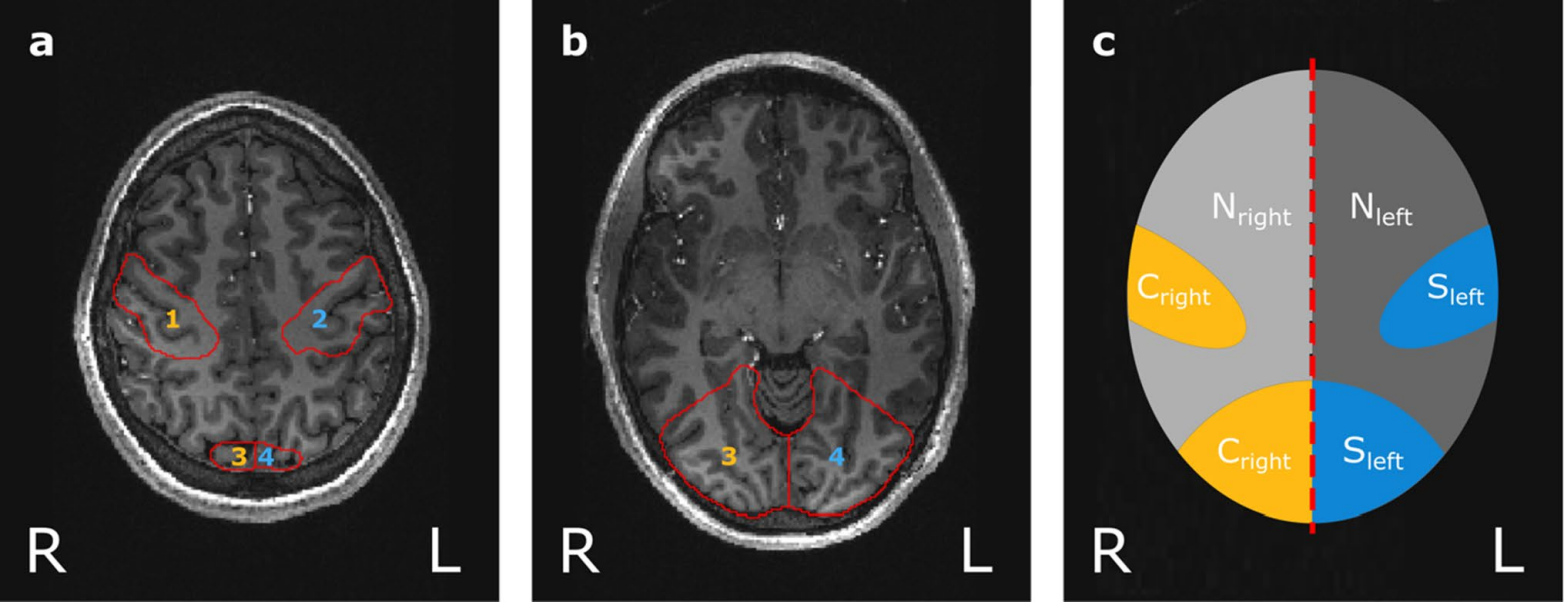
Anatomical data (magnetization prepared rapid gradient echo, MPRAGE) of participant 2 (P2) overlaid with segmentations of sensorimotor and visual cortices, created manually in MITK [17, 18] (German Cancer Research Center, Heidelberg, Germany). **a** Axial slice shows 1: right sensorimotor cortex (negative control), 2: left sensorimotor cortex (stimulated), 3: right visual cortex (negative control), 4: left visual cortex (stimulated). **b** Lower axial slice shows right and left visual cortices (3 and 4). In (**c**) a schema of the regions of interest (ROI) is shown which will be used throughout this article: the blue ROI named stimulated_left_ (S_left_) refers to the stimulated left sensorimotor and visual cortex. The orange ROI control_right_ (C_right_) includes the non-stimulated right sensorimotor and visual cortex and serves as negative control. The non-stimulated area of the left hemisphere is termed non-stimulated_left_ (N_left_), whereas the non-stimulated regions of the right hemisphere are referred to as non-stimulated_right_ (N_right_)

To provide a comparison for the stimulated brain area, a half-sided stimulation paradigm was designed, which combines simultaneous right-sided finger tapping with a half-screen visual paradigm for stimulation of the left sensorimotor and visual cortex, leaving the right cerebral hemisphere as an in-experiment negative control, see Fig. 1.

A block design was used with alternating blocks of 45 s of activation and 15 s of rest, deviating from the classic 30 s/30 s block design which is conventionally used for BOLD experiments [19]. The rationale of this change was to increase the overall "ON"-time of the stimulation paradigm in the experiment to achieve maximal neuronal stimulation. Meanwhile, the 45 s/15 s setup still enables evaluation of the BOLD response, and the resting period of 15 s makes tapping throughout the whole measurement feasible and prevents habituation to the stimulus. This was investigated by conducting two additional BOLD measurements, one without prior stimulation and the second after 40 min of continuous exposition to the stimulation paradigm. The analysis and the BOLD activity maps are shown in the supplements, see Figure S1.

**Fig. 2:**
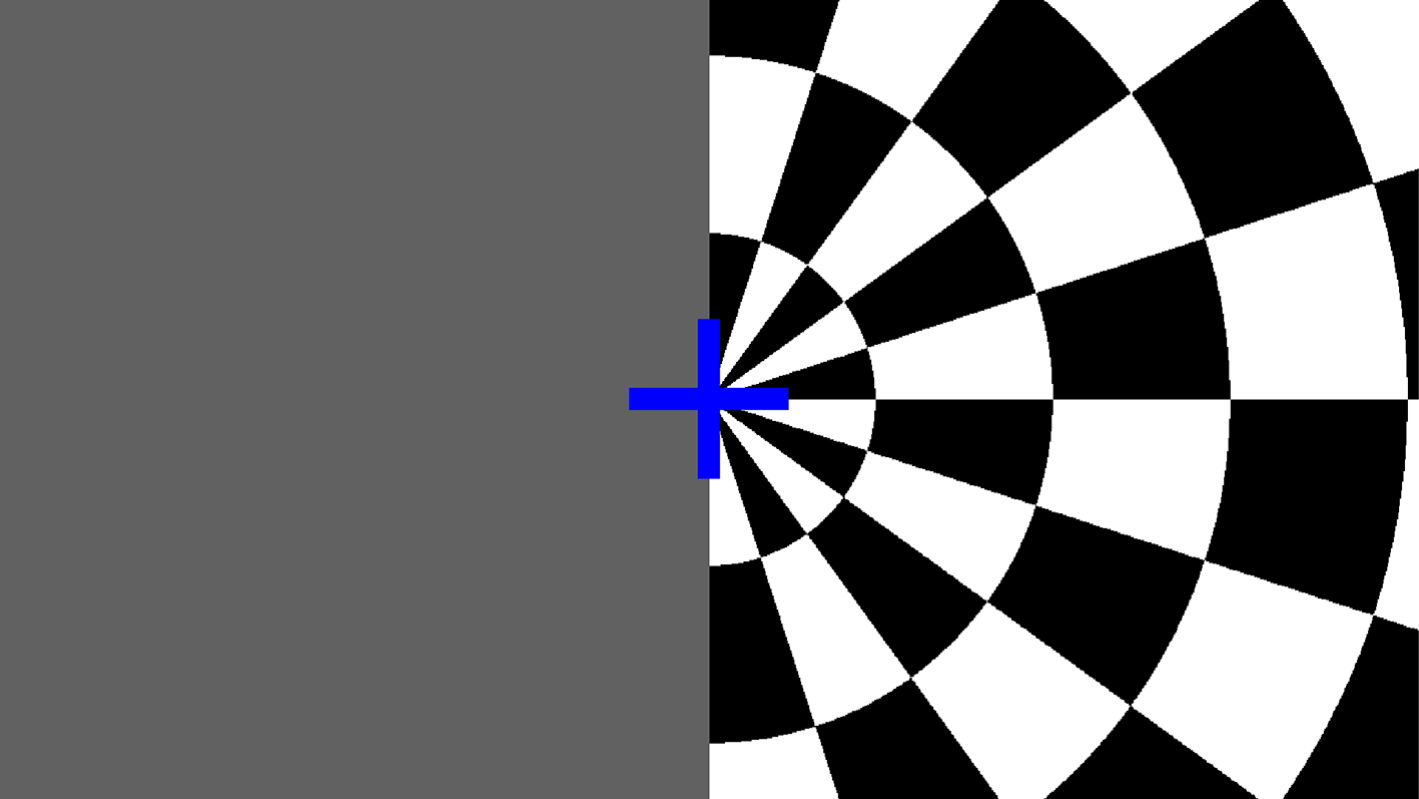
Screenshot of visual paradigm with right-side inversing checkerboard pattern

A commonly used visual stimulus pattern was applied, consisting of a radial checkerboard pattern [20], changing at a rate of 8 Hz [21]. The stimulus was limited to the right-hand side of the screen with iso-luminescent gray on the left side. As attention task, the cross in the center of the screen randomly changes color between blue and red. An exemplary image of the visual paradigm can be seen in Fig. 2. The participant was asked to make a fist with the right hand at every color change of the cross, while being visually observed by the experimenters to check the participant's attention. Additionally, this task was implemented to keep participants focused on the center of the screen.

The right-hand sequential finger-to-thumb tapping was conducted simultaneously with the checkerboard pattern for activation of the sensorimotor cortex.

### Hardware

All examinations were performed on a 7 T whole-body MR system (Magnetom 7 T; Siemens Healthcare, Erlangen, Germany) with horizontal B_0_ orientation and approximately 60 cm bore diameter. The oxygen data were obtained using a home-built ^17^O birdcage head coil with an additional proton (^1^H) channel, used for B_0_ shimming and acquisition of basic proton images [11, 12]. Subsequently, a 24-channel ^1^H head coil (Nova Medical, Wilmington, Massachusetts) was used for acquisition of high-resolution morphological data for registration and segmentation, and for performing the BOLD experiments. For the visual stimulation, an MR-safe screen was set up, which could be viewed from inside the scanner using prism glasses [22].

### MRI protocol

The MRI protocol used for this study consists of three parts: oxygen imaging, BOLD imaging and anatomical imaging. The total acquisition time per measurement amounted to approximately 1.5 h, divided by a 15 min break for coil change.

#### Oxygen imaging

The double-tuned ^17^O/^1^H birdcage coil was used to obtain the oxygen MRI data using a 3D density-adapted radial sequence [23] with a golden angle distribution of projections [24]. In the ^17^O inhalation experiments, 40 min of ^17^O MR data were acquired continuously (TR/TE = 20 ms/0.56 ms, flip angle: 60°, *t*_pulse_ = 1.00 ms, *t*_Readout_ = 5.5 ms, 120 000 projections, number of radial samples = 196, nominal spatial resolution = (7.5 mm)^3^). These parameters were chosen to minimize relaxation weighting and to meet specific absorption rate (SAR) limitations [11]. The experimental inhalation setup is described below. Since the measured ^17^O signal distribution is influenced by transmit and receive profiles of the radiofrequency (RF) coil, the absolute ^17^O images are corrected with a flip angle map [25–27] prior to PV correction. This is not necessary for the relative evaluation of the ^17^O data without PV correction, since the influence of the radiofrequency coils is canceled out. Dynamic ^17^O data were reconstructed with a temporal resolution of ∆t = 1 min and a Hamming filter as well as a zero filling factor of 8 was applied. Additionally, a gradient echo (GRE) image (TR/TE = 7.5 ms/3.25 ms, flip angle: 10°, matrix size: 256 × 256 × 176, nominal resolution: (1 mm)^3^) was acquired with the ^1^H channel of the coil for co-registration of the oxygen data onto high-resolution morphologic data, as described below.

#### Inhalation experiment and H_2_^17^O signal

The oxygen measurement is based on inhalation of the natural occurring, stable ^17^O isotope, which can be measured with MRI in the form of H_2_^17^O after metabolization. A three-phase inhalation experiment was conducted including a baseline, inhalation, and decay phase, during which 3D MR data were acquired continuously as described above. This allows spatially dependent investigation of the ^17^O metabolization.

In the baseline phase, the participant breathes room air (10 min). Here, the ^17^O baseline signal measured in the human brain correlates with the natural abundance of ^17^O_2_ (0.037% [28]). During the inhalation phase, 70%-enriched ^17^O_2_ gas (NUKEM Isotopes GmbH, Alzenau, Germany) is administered to the participant through a breathing system, previously described in detail [11]. The breathing reservoir is filled with approximately 4 L of ^17^O_2_ gas. The inhalation time varies individually and ends when the reservoir is empty (9–12 min). Throughout inhalation of the ^17^O_2_ gas, the signal increase derives from metabolized H_2_^17^O. In the decay phase, a switch back to normal room air is conducted and the measurement is continued, until a total of 40 min of continuous MR data have been acquired. In this phase (duration 18–21 min), the H_2_^17^O produced during the inhalation phase is slowly washed out, and hence the measured oxygen signal starts to decrease. In both the inhalation and decay phase, the signal dynamics vary location-dependently according to the regional metabolic activity. Thus, these differences in metabolism can be investigated by selecting ROI and comparing their signal dynamics.

#### BOLD and anatomical imaging

To ensure that the chosen paradigm stimulates the sensorimotor and visual cortices, BOLD measurements were acquired. An RF coil switch from the ^17^O/^1^H coil to the 24-channel ^1^H head coil allowed the participant a 15 min break within the MRI protocol. BOLD measurements were acquired using the same stimulation paradigm with a T_2_^*^-weighted echo-planar imaging (EPI) sequence (TR/TE = 185 ms/19 ms, flip angle: 12°, 32 slices, isotropic resolution: (2 mm)^3^, TA = 5 s per image, 66 images, 5.5 min total duration). Finally, high-resolution T_1_-weighted anatomical data (magnetization prepared rapid gradient echo, MPRAGE (TR/TE = 3400 ms/1.6 ms, flip angle: 9°, field of view: 262 × 151 × 300 mm^3^, nominal resolution: (0.59 mm)^3^, *T*_Acq_ = 10 min 32 s) were obtained.

### Registration and image segmentation

Registration of the high-resolution anatomical data to the oxygen images was performed in two steps using the FLIRT algorithm of FSL (FMRIB Software Library) [29, 30]. The ^1^H GRE data set was registered to the oxygen images, both acquired with the double-tuned ^17^O/^1^H birdcage coil, using a three parameter model (translation only). Subsequently, the MPRAGE image, acquired with the 24-channel ^1^H head coil, was aligned to the registered GRE data using a six parameter model (translation and rotation). To investigate cerebral gray matter (GM), cerebral white matter (WM) and cerebral spinal fluid (CSF), these three brain regions were obtained by applying the FAST segmentation tool for automatic segmentation to the MPRAGE data set [31]. The 3D masks of sensorimotor and visual cortices were created manually using MITK according to an anatomical atlas [32] in consensus with L.E. and D.P. with 2 and 10 years of experience in neuroimaging. After segmentation, these masks were dilated twice using the MITK dilatation tool to include the spillover due to PV effects. These masks were used for region-wise investigation of the signal evolution in each tissue type. The stimulated ROI comprised the sensorimotor and visual cortex of the left hemisphere, which will be referred to as stimulated_left_ (S_left_) in the following (see Fig. 1c). The sensorimotor and visual cortex of the right hemisphere served as an in-experiment negative control, named control_right_ (C_right_). The non-stimulated ROI included all brain areas excluding the sensorimotor and visual cortex of the left and right hemisphere. These ROI will be referred to as non-stimulated_left_ (N_left_) and non-stimulated_right_ (N_right_). All these regions are also depicted in Fig. 1.

### Data evaluation and statistics

Analogous to the three-phase setup of the inhalation experiment, the H_2_^17^O data curve is divided into three sections: baseline, increase phase and decay phase. A region-wise data evaluation for the stimulated region, its control, and the non-stimulated rest of the brain, each divided into GM and WM, was performed in Matlab (The MathWorks Inc., Natick, USA) with two approaches, one with and one without partial volume (PV) correction:

First, the ^17^O measurement data were converted to H_2_^17^O concentration values using reference bottles filled with regular water. Then, the concentration values were PV corrected [33]. PV correction was performed on the oxygen data set using the tissue masks for GM, WM and CSF, as proposed by Niesporek et al. [33]. This approach furnishes one quantitative PV-corrected H_2_^17^O value within each mask and for each time point. The resulting data curves were then normalized to the baseline. For this, all data points were divided by the mean of the first five baseline values to get relative data. The relative curves can be described with following formula, e.g., for volume S_left_: relative PV-corrected ^17^O signal curve = PV-corrected ^17^O signal (S_left_)/mean(baseline signal values 1–5 (S_left_)). The noise level of the relative PV-corrected H_2_^17^O concentration in GM was estimated by dividing the standard deviation by the mean value of the baseline for P2 (Fig. 4b). In addition, the goodness of the fit was investigated for fitting the model of Atkinson and Thulborn [8] to the PV-corrected data curves of S_left_ and C_right_ for all five participants by calculating the adjusted *R* squared.

Secondly, the signal evolution in each region was determined without PV correction, relative to the baseline. The relative curves were created by spatial averaging of the time evolution of the ^17^O signal within the four volumes S_left_ vs. C_right_ and N_left_ vs. N_right_. Like the PV-corrected data, all data points were divided by the mean of the first five baseline values. This approach eliminates the influences due to inhomogeneities in the transmit and receive profiles.

The resulting data curves of both approaches were compared by their maximal signal entry during the increase phase. To quantify a mean maximal value of S_left_ and C_right_ for each participant 1–5 (P1–5), five data points around TB (switch back to room air) were averaged (called max_P1_ to max_P5_) to reduce noise and compared for the five participant measurements using a one-tailed paired *t* test; see Table 1 for relative PV-corrected data and Table 2 for the relative ^17^O signal evolution. These statistical analyses were performed with Microsoft Excel (version 16.54). For statistical analyses, the level of significance was set to *p* < 0.05.

**Table 1:** Relative evaluation of PV-corrected ^17^O signal (max_P1_ to max_P5_) in the sensorimotor and visual cortices: left (stimulated) vs. right (negative control) for P1–5

	^17^O data stimulated (S_left_)	^17^O data control (C_right_)
Participant		
1	1.27	1.54
2	1.36	1.40
3	1.32	1.21
4	1.35	1.49
5	1.45	1.46
Mean	1.35	1.42
Standard deviation	0.07	0.13
*p* value one-tail	0.168

**Table 2:** Relative ^17^O signal increase (max_P1_ to max_P5_) in the sensorimotor and visual cortices: left (stimulated) vs. right (negative control) for P1–5

	^17^O data stimulated (S_left_)	^17^O data control (C_right_)
Participant		
1	1.32	1.35
2	1.37	1.34
3	1.25	1.23
4	1.36	1.39
5	1.40	1.41
Mean	1.34	1.34
Standard deviation	0.06	0.07
*p* value one-tail	0.333	

The evaluation of the BOLD response was performed using the open-source software SPM 12 [34]. Preprocessing included realignment, smoothing and co-registration with the anatomical image. The statistical analysis in SPM allowed for creation of BOLD activity maps. The *t* value threshold of the activity maps has been set to approximately 5.5 to achieve *p* < 0.05.

## Results

### BOLD measurements

**Fig. 3:**
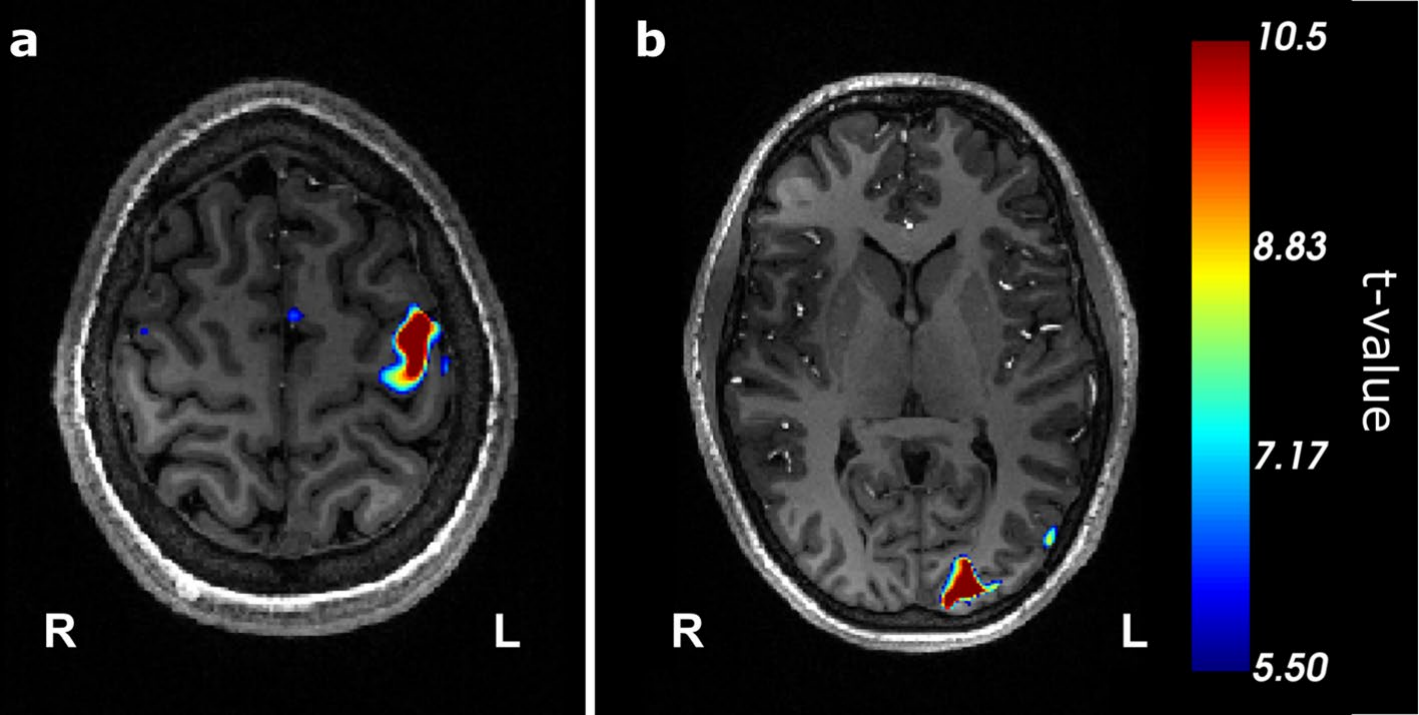
Representative functional MRI data (participant P2) depicting a BOLD map overlaid onto an MPRAGE image showing statistically probable activation within the left sensorimotor (**a**) and left visual cortex (**b**) employing the same paradigm as used for the oxygen experiments

All participants showed significant activity in the stimulated left sensorimotor and visual cortex, while the mirrored areas, serving as negative control, showed hardly any significant activity for a *t* value threshold of 5.5. A resulting BOLD activity map can be seen in Fig. 3. The analysis of the voxel overlap of the significant BOLD activity and ROI used for evaluation of the dynamic ^17^O MRI analysis can be found in the supplements, see Figure S2.

### Relative PV-corrected oxygen data

**Fig. 4:**
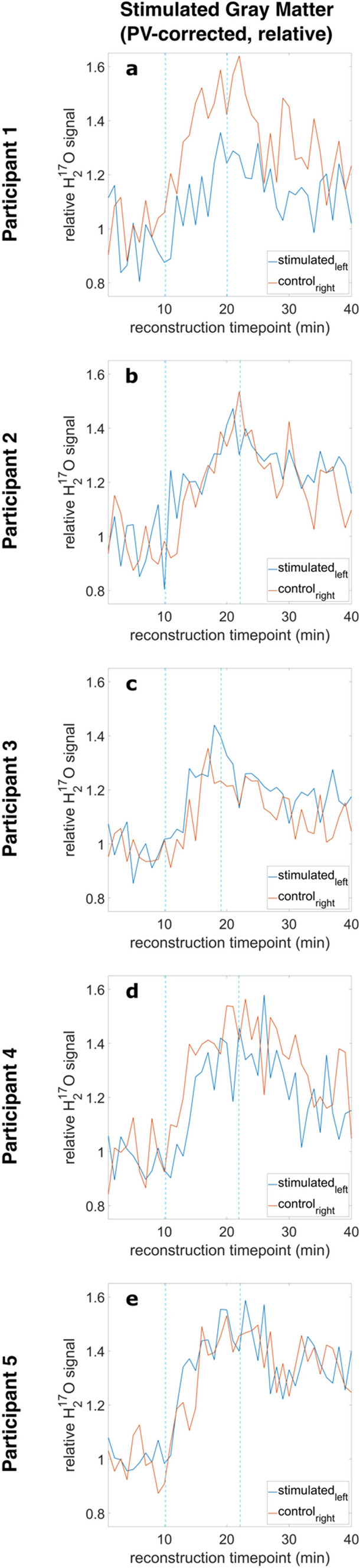
Relative PV-corrected data for all participants (P1–5) of the stimulated GM of the left (S_left_, blue) vs. right (C_right_, control, orange) sensorimotor and visual cortex together. The blue dashed lines indicate beginning and end of ^17^O_2_ gas inhalation. The signal entries show high fluctuation

The flip angle and PV-corrected time evolution of the relative H_2_^17^O concentration of the stimulated GM is depicted in Fig. 4. In the evaluation of GM in the sensorimotor and visual cortex, one volunteer showed a higher signal for the right side in comparison to the left side (Fig. 4a), and one participant showed higher signal entries for S_left_ compared to C_right_ (Fig. 4c). For the other participants, the signals of the right and left stimulated areas do not show a clear trend for either side (Fig. 4b, d, e). However, the noise level leads to major fluctuations of the signal entries in the chosen ROI. The mean value and standard deviation of the baseline H_2_^17^O concentration of P2 (Fig. 4b) was calculated, resulting in a value of (13.2 ± 1.2) mmol/L for the right-sided ROI and (11.7 ± 1.2) mmol/L for the left-sided ROI. Hence, the estimated noise level is 10%, calculated as the coefficient of variation (std/mean). The investigation of the goodness of the fit for the PV-corrected data curves of S_left_ and C_right_ for all five participants yielded adjusted *R* squared values between 0.5 and 0.8.

The PV-corrected evaluation of an even larger ROI, such as the complete GM of the left and right hemisphere (see Figure S3 in the supplements), still shows a mean baseline value and standard deviation of (11.0 ± 0.4 mmol/L) and (11.4 ± 0.6 mmol/L) for left and right side, respectively, from which a noise level of 4.2% was estimated.

These findings motivated the investigation of the relative signal evolutions and the omission of the PV correction to decrease the noise in the signals.

The mean value with standard deviation of the determined maximal values max_P1_ to max_P5_ for the five measurements was 1.35 ± 0.07 for the left stimulated area S_left_, while the resulting mean value with standard deviation for the right-sided control C_right_ was 1.42 ± 0.13. Analyzing these maxima, there was no significant difference between the relative PV-corrected ^17^O signal for the GM in S_left_ and C_right_ (*p* = 0.168), see Table 1.

### Relative oxygen signal evolution

**Fig. 5:**
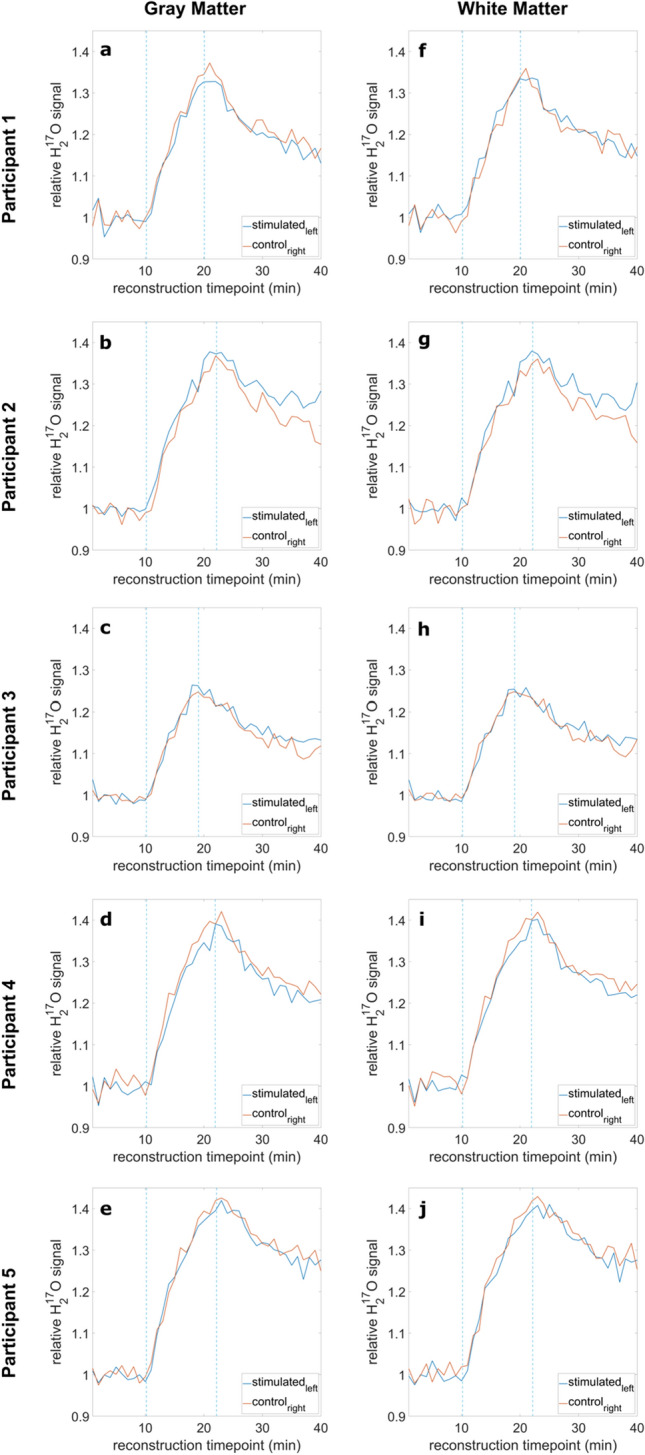
Relative ^17^O signal evolutions for all participants (P1–5) for GM (**a**–**e**) and WM (**f**–**j**) of the left vs. right sensorimotor and visual cortex together (S_left_ vs. C_right_). No major differences were visible in comparison between S_left_ and C_right_ for all participants

In the following, the relative H_2_^17^O signals for the stimulated GM and WM are compared for all five participants without PV correction, as depicted in Fig. 5. For the stimulated GM, two experiments visually showed a higher maximal signal for the left side (S_left_), (Fig. 5b, c). For participant P2 in particular, S_left_ showed a steeper slope during the inhalation phase with higher overall maximum compared to C_right_, with diverging curves especially during the decay phase. However, two other experiments resulted in higher maxima for C_right_ (Fig. 5a, d). One experiment did not show major differences between right and left side (Fig. 5e). For the stimulated WM (Fig. 5f–j), similar observations were made as for the stimulated GM, but with fewer differences between S_left_ and C_right_. The lower maximum in relative H_2_^17^O signal for P3 compared to the other experiments is due to a shorter ^17^O_2_ inhalation time.

**Fig. 6:**
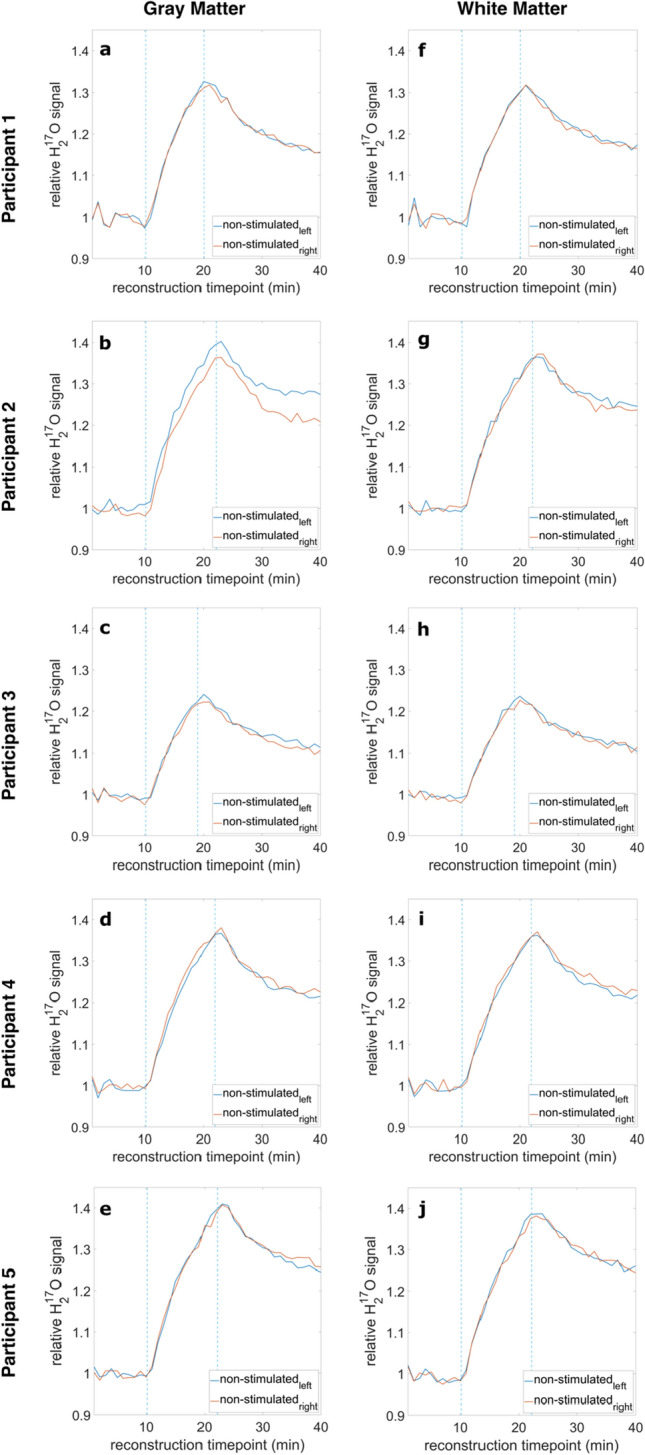
Relative signal evolutions for all participants (P1–5) for GM (**a**–**e**) and WM (**f**–**j**) of the non-stimulated brain areas (N_left_ vs. N_right_). Most of the experiments showed only slight differences between N_left_ and N_right_. In P2, the non-stimulated GM shows a particularly high difference between N_left_ and N_right_

The relative H_2_^17^O signal evolution of the non-stimulated GM and WM is shown in Fig. 6 for all five participants. In three experiments, the non-stimulated GM showed higher maximal signal entries for the left side (N_left_) (Fig. 6a–c). The results for P2 (Fig. 6b) show a particularly high difference between N_left_ and N_right_ compared to the other participants. For one participant, the data of the right side (N_right_) were slightly higher in comparison to the left side (Fig. 6d), and one experiment showed no major difference (Fig. 6e). Similar results were obtained for the non-stimulated white matter: N_left_ showed higher maximal values in two measurements (Fig. 6h, j), one measurement did not display any difference between left and right (Fig. 6f), and in two experiments N_right_ exhibited higher signal entries compared to N_left_ (Fig. 6g, i).

The mean value with standard deviation of the determined maximal values max_P1_ to max_P5_ for the five measurements was 1.34 ± 0.06 for the left stimulated ROI S_left_, while the resulting mean value with standard deviation for the right-sided control C_right_ was 1.34 ± 0.07. Statistical evaluation was performed using a paired one-tailed *t* test of the ^17^O signal in the stimulated left sensorimotor and visual cortex and the right sensorimotor and visual cortex serving as negative control. There was no significant difference between the ^17^O signal in the left (stimulated) and right (negative control) sensorimotor and visual cortex (*p* = 0.333), as can be seen in Table 2.

## Discussion

The aim of this study was to test the feasibility of detecting neuronal activity employing dynamic ^17^O MRI.

The BOLD experiments showed significant stimulation of the left sensorimotor and visual cortex in all five participants, thus validating the paradigm chosen for this study. The relative PV-corrected oxygen data showed high signal fluctuations, impeding further quantitative analysis and valid interpretation. Oxygen imaging did not show significant differences in the relative signal evolution of H_2_^17^O signal between the stimulated left sensorimotor and visual cortex and the right-sided negative control in this study.

### Data analysis

#### BOLD measurements

The BOLD measurements, which were performed after the dynamic ^17^O MRI, served as a control for the oxygen experiments to show that the paradigm did indeed stimulate the investigated ROI. The BOLD activity maps showed that significant stimulation of the left sensorimotor and left visual cortex was achieved for all five experiments, validating the employed paradigm as well as the selected ROI.

#### Oxygen imaging

The oxygen data were analyzed using two different approaches. Firstly, the data were corrected for PV effects, since they pose a major challenge in ^17^O MRI [12, 33] and the correction for PV effects is a possibility to reduce bias. The data were then normalized to the baseline. However, the resulting curves for the relative PV-corrected stimulated GM, which corresponds to a small ROI, showed high signal fluctuations. The goodness of the fit, determined by calculating the adjusted *R* squared, was below 0.9 for the PV-corrected data curves of all five participants, which, in general, is considered the cutoff for an acceptable data fit. The baseline, which ideally is supposed to be constant, shows a noise level of approximately 10%. By choosing a larger ROI, the local change in oxygen metabolism is diluted even more, thus compromising the possibility of detecting neuronal activity. But even when looking at larger ROI with a higher signal-to-noise ratio (SNR), e.g., the complete GM of the left hemisphere (see Figure S3), a noise level of about 4.2% remains. Hence, in the presented setup the noise in PV-corrected data impedes detecting changes in the oxygen signal evolution arising from regionally elevated metabolic activity.

Secondly, the relative signal evolution of the oxygen data without PV correction was investigated. The relative signal evolution resulted in heterogenous data for the five experiments. For the stimulated GM, no trend in favor of the left or right side could be determined. Two measurements showing higher maximal values for the left stimulated brain areas seemed to fit the established considerations, which assume neuronal activity can be detected employing dynamic ^17^O MRI. Especially experiment P2 seemed to show a great difference between the left and right sensorimotor and visual cortex, matching the assumptions made in advance. However, two other experiments yielded opposite results, as seen for instance in experiment P5. The observed differences in signal characteristics for the stimulated GM were also seen in the non-stimulated GM (compare Figs. 5b and 6b). In addition, no significant difference between the determined maximal value of the relative ^17^O signal in the stimulated left versus the non-stimulated right sensorimotor and visual cortex was found.

From this, we infer that the observed changes in the oxygen data were rather due to internal signal fluctuations caused by noise and other influences than from external stimulation. This in turn leads to the conclusion that neuronal activity cannot be detected employing our experimental setup of dynamic ^17^O MRI at 7 T.

### Limitations

This study has several limitations, which have to be considered. The main issue employing dynamic ^17^O imaging is the low SNR. For the chosen ROI, the relative PV-corrected signal evolution of the oxygen data is dominated by noise and thus was not used for further quantitative analysis or interpretation. As seen in initial experiments, the noise level also impeded the quantitative determination of CMRO_2_. Instead, we evaluated the relative ^17^O signal evolution of the dynamic H_2_^17^O signal without PV correction, which is intrinsically corrected for inhomogeneities in the transmit and receive profiles.

Secondly, neuronal stimulation in the human brain has not yet been studied employing dynamic ^17^O MRI, so the effect size caused by the stimulation in healthy volunteers was not known in advance. In animal models functional ^17^O MR studies have demonstrated promising results. Zhu et al. conducted a study in which six cats underwent functional ^17^O MRS at 9.4 T with visual stimulation [15]. The results showed a 32.3% ± 10.8% increase of CMRO_2_ in the activated visual cortex V1, while the surrounding brain regions exhibited a decrease of CMRO_2_ [15]. The net CMRO_2_ increase induced by visual stimulation was found to be 9.7% ± 1.9% after spatial averaging [15]. Our study investigating healthy volunteers using dynamic ^17^O MRI did not reveal a significant difference between the left, stimulated visual and sensorimotor cortex S_left_ and the contralateral control C_right_. There are several points to consider when comparing these two studies. For one, the anatomical differences between the human and cat brain must be taken into account. For instance, cats have a 3.5-fold higher GM/WM ratio compared to humans [35]. PV effects might have a larger influence in the human brain, as the WM "spillover" could have a more significant diluting effect on the stimulated GM. Furthermore, we used the Atkinson and Thulborn model for data analysis in this study, which was adapted especially for the use in humans and does not rely on the experimental determination of the cerebral blood flow [8]. Recently, sensory stimulation has been investigated in a rat model, using the same Atkinson and Thulborn model for data analysis [36]. The abstract presented results of functional ^17^O MR experiments at 11.7 T in which rats were exposed to electrostimulation. Here, a 7% ± 5% CMRO_2_ increase is reported [36].

So far, no studies employing functional ^17^O MRI are available for the application in humans. However, in 2014 Zhu et al. reported preliminary results of the effect of a visual stimulus paradigm using ^17^O MRS on the human occipital lobe in one subject [16]. Here, a > 10% increase of CMRO_2_ was detected [16].

Other neuroimaging methods, calibrated BOLD imaging and oxygen-15 (^15^O) positron emission tomography (PET), were researched to estimate the possible effect of the paradigm. For calibrated BOLD experiments using finger tapping tasks, literature reports CMRO_2_ increases in the sensorimotor cortex between 13% [37] and 30% [38]. Reported changes in CMRO_2_ for ^15^O PET stimulation studies were in the order of 5% for sensorimotor stimulation [39] and between 5 and 15% for visual stimulation, depending on the duration of stimulation, with higher results for longer exposition to the visual stimulus [40]. Other indirect oximetry approaches such as QUantitative Imaging of eXtraction of Oxygen and TIssue Consumption (QUIXOTIC) [41] resulted in significant changes to the oxygen metabolism after visual stimulation.

Thus, a range of 5–30% change to the local oxygen metabolism during external visual or sensorimotor stimulation is reported. Consequently, we assumed that the stimulation paradigm used in our study achieves a similar change in the local oxygen turnover. To achieve a large stimulation region we combined a sensorimotor and visual task. However, there could be other paradigms that might achieve a stimulation of an even greater brain area.

Furthermore, the study was performed with a relatively small number of participants (*n* = 5). The main reason for the small number of participants is the high costs of ^17^O_2_ gas. Yet, the sample size is within the typical range for technical development studies [42]. As the real effect size was not known in advance, we performed a post hoc power analysis to estimate the needed sample size for different noise levels (5% and 10%) and the two limits of assumed change in CMRO_2_, 5% and 30%. The detailed analysis can be found in the supplements. Investigating the maximum and assuming that the model perfectly described the signal course in vivo and that there were no further influences, it would have been theoretically possible to detect a change in the maximum for a 30% CMRO_2_ change, despite the noise level of 10% in the signal course and the small sample size of *n* = 5. With these conditions we estimated that our study had enough power (power = 0.8) to detect CMRO_2_ changes $\ge$ 23%. However, since other influences can affect the evaluation, such as incorrect segmentation or registration of the ^1^H data, the calculated sample sizes and minimal detectable CMRO_2_ change are probably only lower limits and can be even higher. In case the changes in CMRO_2_ induced by the stimulation paradigm were small (e.g., 5%), this study is underpowered and would not have been able to detect a significant difference. Hence, it would be interesting to repeat the study with a larger number of participants, especially if technical optimizations are available in future, reducing the noise level.

In addition, the experimental setup for this study is complex and requires profound knowledge of the technology. Dynamic ^17^O imaging requires specialized coil equipment and benefits from a higher B_0_ field strength as it increases the signal to noise ratio. These technical prerequisites are not available at many research sites, complicating the applicability of the imaging modality.

These factors explain the limited amount of literature on dynamic ^17^O imaging and the lack of similar studies for comparison. However, recently an RF coil was presented that allows, for example, ^17^O MRI and high-resolution structural brain ^1^H MRI at 3 T [43], and some hardware parts were also optimized so that it could also be used for comodal PET.

An increase in field strength, for example 14.0 T MRI, as well as the use of dedicated coils (e.g., additional receive array or less SAR-restrictive transmit coil) could increase the SNR, improve the resolution of dynamic ^17^O MRI and may render the detection of neuronal stimulation possible. While the availability of ultrahigh field MRI scanners is still limited, it is increasing steadily since the FDA approval for certain clinical applications in 2017 [44]. Furthermore, a trend toward research studies at even higher field strengths such as 9.4 T, 10.5 T, 11.7 T, or 14.0 T is expected [45, 46].

## Conclusion

In conclusion, this study showed that the change of cerebral oxygen metabolism induced by sensorimotor and visual stimulation could not be resolved employing the current setup and methodology of dynamic ^17^O MRI at 7 T. Accordingly, this study suggests that potential clinical ^17^O MRI studies are not biased by external stimuli during the measurement. Hence, the strength of dynamic ^17^O MRI with the current setup and field strength does not lie in the investigation of physiological changes to the cerebral oxygen metabolism induced by external stimulation (e.g., sensorimotor and visual stimulation), but rather in the investigation of cerebral pathologies with larger impact on the oxygen metabolism. In addition to research applications in tumors [12, 13], promising applications of the modality include neurodegenerative diseases such as Alzheimer’s dementia [47, 48], Parkinson’s disease [49], and especially stroke imaging [50].

### Supplementary Information

Below is the link to the electronic supplementary material.

Supplementary file1 (DOCX 4168 KB)

## Data Availability

In accordance with the institutional review board, the data acquired in this study contain person-sensitive information, which can be shared only in the context of scientific collaborations.

## References

[1] Glover GH (2011). Overview of functional magnetic resonance imaging. Neurosurg Clin N Am.

[2] Scarapicchia V, Brown C, Mayo C, Gawryluk JR (2017). Functional magnetic resonance imaging and functional near-infrared spectroscopy: insights from combined recording studies. Front Hum Neurosci.

[3] Gauthier CJ, Fan AP (2019). BOLD signal physiology: models and applications. Neuroimage.

[4] Matthews PM, Honey GD, Bullmore ET (2006). Applications of fMRI in translational medicine and clinical practice. Nat Rev Neurosci.

[5] Hillman EM (2014). Coupling mechanism and significance of the BOLD signal: a status report. Annu Rev Neurosci.

[6] Arthurs OJ, Boniface S (2002). How well do we understand the neural origins of the fMRI BOLD signal?. Trends Neurosci.

[7] Gordji-Nejad A, Mollenhoff K, Oros-Peusquens AM, Pillai DR, Shah NJ (2014). Characterizing cerebral oxygen metabolism employing oxygen-17 MRI/MRS at high fields. MAGMA.

[8] Atkinson IC, Thulborn KR (2010). Feasibility of mapping the tissue mass corrected bioscale of cerebral metabolic rate of oxygen consumption using 17-oxygen and 23-sodium MR imaging in a human brain at 9.4 T. Neuroimage.

[9] Hoffmann SH, Begovatz P, Nagel AM, Umathum R, Schommer K, Bachert P, Bock M (2011). A measurement setup for direct 17O MRI at 7 T. Magn Reson Med.

[10] Borowiak R, Groebner J, Haas M, Hennig J, Bock M (2014). Direct cerebral and cardiac 17O-MRI at 3 Tesla: initial results at natural abundance. MAGMA.

[11] Niesporek SC, Umathum R, Lommen JM, Behl NGR, Paech D, Bachert P, Ladd ME, Nagel AM (2018). Reproducibility of CMRO2 determination using dynamic (17) O MRI. Magn Reson Med.

[12] Hoffmann SH, Radbruch A, Bock M, Semmler W, Nagel AM (2014). Direct (17)O MRI with partial volume correction: first experiences in a glioblastoma patient. MAGMA.

[13] Paech D, Nagel AM, Schultheiss MN, Umathum R, Regnery S, Scherer M, Wick A, Platt T, Wick W, Bendszus M, Unterberg A, Schlemmer HP, Ladd ME, Niesporek SC (2020). Quantitative dynamic oxygen 17 MRI at 7.0 T for the cerebral oxygen metabolism in glioma. Radiology.

[14] Ebersberger L, Kratzer FJ, Potreck A, Niesporek SC, Keymling M, Nagel AM, Bendszus M, Wick W, Ladd ME, Schlemmer H-P, Hoffmann A, Platt T, Paech D (2023). First application of dynamic oxygen-17 magnetic resonance imaging at 7 Tesla in a patient with early subacute stroke. Front Neurosci.

[15] Zhu XH, Zhang N, Zhang Y, Ugurbil K, Chen W (2009). New insights into central roles of cerebral oxygen metabolism in the resting and stimulus-evoked brain. J Cereb Blood Flow Metab.

[16] Zhu XH, Liu X, Lu M, Wiesner HM, Ugurbil K, Chen W (2014) In vivo 17O MR imaging and quantification of CMRO2, CBF and OEF in human visual cortex at rest and during activation. In: Proc ISMRM #3763

[17] Nolden M, Zelzer S, Seitel A, Wald D, Muller M, Franz AM, Maleike D, Fangerau M, Baumhauer M, Maier-Hein L, Maier-Hein KH, Meinzer HP, Wolf I (2013). The medical imaging interaction toolkit: challenges and advances: 10 years of open-source development. Int J Comput Assist Radiol Surg.

[18] Wolf I, Vetter M, Wegner I, Bottger T, Nolden M, Schobinger M, Hastenteufel M, Kunert T, Meinzer HP (2005). The medical imaging interaction toolkit. Med Image Anal.

[19] Kao MH, Temkit M, Wong WK (2014). Recent developments in optimal experimental designs for functional magnetic resonance imaging. World J Radiol.

[20] Huettel SA, McCarthy G (2000). Evidence for a refractory period in the hemodynamic response to visual stimuli as measured by MRI. Neuroimage.

[21] Singh M, Kim S, Kim TS (2003). Correlation between BOLD-fMRI and EEG signal changes in response to visual stimulus frequency in humans. Magn Reson Med.

[22] Groebner J, Berger MC, Umathum R, Bock M, Rauschenberg J (2013). 7 Tesla compatible in-bore display for functional magnetic resonance imaging. MAGMA.

[23] Nagel AM, Laun FB, Weber MA, Matthies C, Semmler W, Schad LR (2009). Sodium MRI using a density-adapted 3D radial acquisition technique. Magn Reson Med.

[24] Chan RW, Ramsay EA, Cunningham CH, Plewes DB (2009). Temporal stability of adaptive 3D radial MRI using multidimensional golden means. Magn Reson Med.

[25] Allen SP, Morrell GR, Peterson B, Park D, Gold GE, Kaggie JD, Bangerter NK (2011). Phase-sensitive sodium B1 mapping. Magn Reson Med.

[26] Morrell GR (2008). A phase-sensitive method of flip angle mapping. Magn Reson Med.

[27] Insko EKBL (1993). Mapping of the radiofrequency field. J Magn Reson A.

[28] Nier AO (1950). A redetermination of the relative abundances of the isotopes of carbon, nitrogen, oxygen, argon, and potassium. Phys Rev.

[29] Jenkinson M, Bannister P, Brady M, Smith S (2002). Improved optimization for the robust and accurate linear registration and motion correction of brain images. Neuroimage.

[30] Jenkinson M, Smith S (2001). A global optimisation method for robust affine registration of brain images. Med Image Anal.

[31] Zhang Y, Brady M, Smith S (2001). Segmentation of brain MR images through a hidden Markov random field model and the expectation-maximization algorithm. IEEE Trans Med Imaging.

[32] Schünke M, Schulte E, Schumacher U, Voll M, Wesker K (2018). Prometheus Lernatlas-Kopf, Hals und Neuroanatomie.

[33] Niesporek SC, Hoffmann SH, Berger MC, Benkhedah N, Kujawa A, Bachert P, Nagel AM (2015). Partial volume correction for in vivo (23)Na-MRI data of the human brain. Neuroimage.

[34] Friston KJ, Holmes AP, Worsley KJ, Poline JP, Frith CD, Frackowiak RS (1994). Statistical parametric maps in functional imaging: a general linear approach. Hum Brain Mapp.

[35] Zhu XH, Qiao H, Du F, Xiong Q, Liu X, Zhang X, Ugurbil K, Chen W (2012). Quantitative imaging of energy expenditure in human brain. Neuroimage.

[36] Tourais A, Hery C, Guillermier M, Valette J, Baligand C (2023) 17O-MRI of the cerebral metabolic rate of oxygen during electrical stimulation of the paws in the rat at 11.7 T. In: Proc ISMRM #4723

[37] Yucel MA, Huppert TJ, Boas DA, Gagnon L (2012). Calibrating the BOLD signal during a motor task using an extended fusion model incorporating DOT, BOLD and ASL data. Neuroimage.

[38] Guidi M, Huber L, Lampe L, Gauthier CJ, Moller HE (2016). Lamina-dependent calibrated BOLD response in human primary motor cortex. Neuroimage.

[39] Fox PT, Raichle ME (1986). Focal physiological uncoupling of cerebral blood flow and oxidative metabolism during somatosensory stimulation in human subjects. Proc Natl Acad Sci U S A.

[40] Mintun MA, Vlassenko AG, Shulman GL, Snyder AZ (2002). Time-related increase of oxygen utilization in continuously activated human visual cortex. Neuroimage.

[41] Stout JN, Adalsteinsson E, Rosen BR, Bolar DS (2018). Functional oxygen extraction fraction (OEF) imaging with turbo gradient spin echo QUIXOTIC (Turbo QUIXOTIC). Magn Reson Med.

[42] Hanspach J, Nagel AM, Hensel B, Uder M, Koros L, Laun FB (2021). Sample size estimation: current practice and considerations for original investigations in MRI technical development studies. Magn Reson Med.

[43] Lakshmanan K, Dehkharghani S, Madelin G, Brown R (2020). A dual-tuned (17) O/(1) H head array for direct brain oximetry at 3 Tesla. Magn Reson Med.

[44] Administration USFD (2017) FDA clears first 7T magnetic resonance imaging device. Accessed 13 Feb 2022

[45] Platt T, Ladd ME, Paech D (2021). 7 Tesla and beyond: advanced methods and clinical applications in magnetic resonance imaging. Invest Radiol.

[46] Budinger TF, Bird MD, Frydman L, Long JR, Mareci TH, Rooney WD, Rosen B, Schenck JF, Schepkin VD, Sherry AD, Sodickson DK, Springer CS, Thulborn KR, Ugurbil K, Wald LL (2016). Toward 20 T magnetic resonance for human brain studies: opportunities for discovery and neuroscience rationale. MAGMA.

[47] Maurer I, Zierz S, Moller HJ (2000). A selective defect of cytochrome c oxidase is present in brain of Alzheimer disease patients. Neurobiol Aging.

[48] Wong-Riley M, Antuono P, Ho KC, Egan R, Hevner R, Liebl W, Huang Z, Rachel R, Jones J (1997). Cytochrome oxidase in Alzheimer's disease: biochemical, histochemical, and immunohistochemical analyses of the visual and other systems. Vis Res.

[49] Beal MF (1992). Does impairment of energy metabolism result in excitotoxic neuronal death in neurodegenerative illnesses?. Ann Neurol.

[50] Delapaz R, Gupte P (2011). Potential application of (1)(7)O MRI to human ischemic stroke. Adv Exp Med Biol.

