# Supplementary material for: First implementation of dynamic oxygen-17 (17O) magnetic resonance imaging at 7 Tesla during neuronal stimulation in the human brain
Source: MAGMA. 2023 Sep 22;37(1):27–38. doi: 10.1007/s10334-023-01119-6 (PMC10876824; doi:10.1007/s10334-023-01119-6)
Supplement: Supplementary file 1 — Supplementary file1 (DOCX 4168 KB) [file 10334_2023_1119_MOESM1_ESM.docx]

Supplementary Material

First implementation of dynamic oxygen-17 (^17^O) magnetic resonance imaging at 7 Tesla during neuronal stimulation in the human brain

Louise Ebersberger^1,2,3^, Fabian J. Kratzer^4^, Vanessa L. Franke^4,5^, Armin M. Nagel^4,6^, Sebastian C. Niesporek^4^, Andreas Korzowski^4^, Mark E. Ladd^2,4,5^, Heinz-Peter Schlemmer^1^, Daniel Paech^1,7^, Tanja Platt^4^

^1^ German Cancer Research Center (DKFZ) Heidelberg, Division of Radiology, Heidelberg, Germany.

^2^ Faculty of Medicine, Ruprecht-Karls University Heidelberg, Heidelberg, Germany.

^3^ Department of Pediatrics, Bern University Hospital, Bern, Switzerland.

^4^ German Cancer Research Center (DKFZ) Heidelberg, Division of Medical Physics in Radiology, Heidelberg, Germany.

^5^ Faculty of Physics and Astronomy, Ruprecht-Karls University Heidelberg, Heidelberg, Germany.

^6^ Institute of Radiology, Friedrich-Alexander University Hospital Erlangen-Nürnberg (FAU), University Hospital Erlangen, Erlangen, Germany.

^7^ Department of Neuroradiology, University Hospital Bonn, Bonn, Germany.

**Corresponding author:**

Dr. Tanja Platt

German Cancer Research Center (DKFZ)

Im Neuenheimer Feld 280

69120 Heidelberg

Germany

t.platt@dkfz-heidelberg.de

Tel.: +49 6221-42 3066

Fax: +49 6221-42 2585

**Acknowledgements**

The authors thank Dr. Wietske van der Zwaag and Dr. Natalia Petridou for sharing their expertise on fMRI. Furthermore, the authors thank NUKEM Isotopes GmbH (Alzenau, Germany) for their supply with ^17^O_2_ gas at a reduced cost.

# Supplementary Data

**Influence of time on BOLD activity induced by combined sensorimotor-visual paradigm**

To check for possible habituation effects, the influence of applying the stimulation paradigm over a period of 40 min was investigated. For this, P5 underwent two additional BOLD measurements: the first was acquired without any prior stimulation, like the previous BOLD experiments used as a control for the dynamic ^17^O MRI experiments. The second BOLD was measured 40 min after the first. In between the two measurements, the volunteer was continuously exposed to the visual stimulation paradigm and kept on finger tapping.

The two BOLD maps showing significant activity (t-value > 5.5) are compared in Figure S1. Subfigures a)-d) show the data set without prior stimulation, while e)-h) show the BOLD data after 40 min of continuous right-sided visual stimulation and right-handed finger tapping.

The results show similar activity in the sensorimotor cortex for both measurements. The visual stimulation yields sufficient, but slightly reduced number of significant voxels after 40 min (Fig. S1 f)) compared to the measurement without prior stimulation (Fig. S1 b)).

The additional BOLD measurements suggest that the chosen paradigm in this study yields sufficient stimulation over 40 min of continuous exposition. Possible habituation effects might have been prevented by the implemented attention task.

**Evaluation of voxel overlap of significant BOLD activity and ROI used for dynamic ^17^O MRI analysis**

Figure S2 shows an MPRAGE image of P5 with the ROI of the left sensorimotor and visual cortex used for data analysis of the dynamic ^17^O MRI data set in white, overlaid with the BOLD activity map. For evaluation of the overlap, the two volumes were compared visually (see Figure S2), and quantitatively. Visually, the two volumes show a good overlap. However, the white ROI is larger than the region of significant BOLD signal. In numbers, the white ROI used for data analysis of the dynamic ^17^O data comprises 148114 voxels, while the BOLD area consists of 10508 voxels. The overlap of the two volumes are 10317 voxels. This is equivalent to 7% of the white ROI.

The evaluation of the two volumes shows, that the overlap is satisfactory. However, the ROI for the left sensorimotor and visual cortex used for analysis of the oxygen data set is notably larger than the overlaid BOLD activity. The segmentation of the left sensorimotor and visual cortex was a trade-off between precision and volume size, as the effective resolution of dynamic ^17^O MRI is quite low due to a low signal-to-noise ratio. The nominal resolution of dynamic ^17^O MRI is (7.5mm)^3^, but considering the spillover effects due to the point spread function (PSF), the effective resolution is worse; the simulated full width half maximum of the PSF is approximately 2.3 voxels, resulting in an effective resolution of the oxygen images of about ((17-17.5)mm)³ for WM and GM [1]. The data analysis using PV correction was already not possible using the larger, white ROI, as described in the manuscript. Since the BOLD volume would comprise even less than the white ROI, data analysis using the smaller BOLD ROI was not performed.

**Influence of noise on the dynamic ^17^O signal course and sample size estimation**

In this study, we estimated a noise level of about 10% in the PV-corrected signal evolution for the chosen ROI S_left_ (equivalent to the stimulated left sensorimotor and visual cortex, see Fig. 1c in the manuscript) for the used experimental setup. Furthermore, the real effect size of the stimulation paradigm was not known prior to conducting the study, but literature for functional ^17^O MR animal stimulation studies and preliminary results in one volunteer for a visual stimulus, as well as comparable metabolic neuroimaging techniques with stimulation paradigm in humans suggested a change in CMRO_2_ between 5% and 30% (see also discussion in the manuscript).

The goal of this additional analysis was to i) investigate the influence of noise on the dynamic ^17^O signal course, and ii) estimate the minimum number of participants necessary to achieve a given power (sample size) for the two limits, 5% and 30% CMRO_2_ change, for two different analysis approaches: firstly, CMRO_2_ estimation (not reported in this manuscript due to high noise level), and secondly, the investigation of the maximum of the dynamic ^17^O signal curve (see result section 3.2 in the manuscript).

For this, normally distributed random noise with a standard deviation of 5% and 10% was added to the model from Atkinson and Thulborn [2] for various input CMRO_2_ values, and the output CMRO_2_ values were evaluated by fitting the model to the curve. The input values were chosen as 2.31 µmol/(g min) as "baseline" CMRO_2_ value without stimulation (0% CMRO_2_ change; control), as reported in a previous study [3]. The values of 2.43 and 3.00 µmol/(g min) correspond to +5% and +30% CMRO_2_ change (simulated stimulation), respectively. Then, the fitted CMRO_2_ and maximum value for the signal course was investigated for 1000 different random noise simulations. Mean values, standard deviations, cohen’s d values and calculated sample sizes are summarized in table S1. Cohen’s d describes a standardized effect size and is given here as an orientation value (cohen’s d of 0.20: small effect size, 0.50: medium, 0.80: large, 1.2: very large, 2.0: huge) [4,5]. The sample size was calculated using the resulting cohen’s d with following conditions: one-tailed paired t-test, alpha=0.05, power=0.8.

As can be seen from table S1, the effect sizes for the maximum are larger than for the fitted CMRO_2_ value. Therefore, a change in the maximum value should be more reliably detectable than in the CMRO_2_ value. Thus, we decided to investigate only the maximum value quantitatively in our stimulation study. For a 30% change in CMRO_2_ value from 2.31 to 3.00 µmol/(g min), high effect sizes result for the fitted CMRO_2_ and the determined maximum for 5% noise (cohen’s d of 1.18 and 3.89, calculated sample sizes: n= 7 and n=3, respectively). For 10% noise, only the effect size for a change in the maximum value is high for a 30% CMRO_2_ change (cohen’s d: 1.99, calculated sample size: n=4). A change of 5% in CMRO_2_ from 2.31 to 2.43 µmol/(g min) results in a medium effect size for the maximum value (0.59) for 5% noise and a small effect size (0.32) for 10 % noise. Here, cohen’s d is below 0.2 for the fitted CMRO_2_ value.

The necessary sample sizes to achieve a given power of 0.8 via analyzing the maximum value for a 30% CMRO_2_ change (both 5% or 10% noise) are lower than our number of participants in this study of n = 5. If the model perfectly described the signal course in vivo and if there were no further influences, the conclusion might be that 5 participants would be sufficient to identify a significant change with a power of 0.8 in these cases. However, since other influences can affect the evaluation, such as incorrect segmentation or registration of the ^1^H data, the calculated size is probably only a lower limit and is probably even higher.

Assuming, on the other hand, the induced change in CMRO_2_ is only 5%, our study would be underpowered, for both noise levels, and to achieve sufficient power for an ideal model and signal evolution, 20 participants would have been needed for a noise level of 5%, and 62 volunteers for a noise level of 10%.

We estimated which change in CMRO_2_ would have theoretically been feasible to detect using our current experimental setup with approximately 10% noise in the signal course for the chosen ROI and a sample size of 5 participants: we found that for investigating the maximum, our study theoretically had sufficient power (power=0.8) to detect changes in CMRO_2_ of 23%. However, due to the reasons mentioned above, this is only an estimate of a lower detection limit and might be even higher.

**Table S1: Mean values, standard deviations, cohen’s d values and calculated sample sizes (minimum numbers of participants necessary to achieve a power of 0.8) for 1000 simulations with normally distributed random noise with a standard deviation of 5% and 10%, which was added to the model from Atkinson and Thulborn.** For the fitted CMRO_2_ value and the determined maximum value also the cohen’s d values are given for comparing the distributions for a given $\Delta$ CMRO_2_ with the distribution for an input CMRO_2_ value of 2.31 µmol/(g min), as well as the calculated sample sizes.

| **Input parameters** |  |  |  |  |  |  |  |  |
| --- | --- | --- | --- | --- | --- | --- | --- | --- |
| Noise | 5% | 5% | 5% | 10% | 10% | 10% |  | 10% |
| Input CMRO_2_ in µmol/(g min) | 2.31 | 2.43 | 3.00 | 2.31 | 2.43 | 3.00 |  | 2.84 |
| $\Delta$ CMRO_2_ in % from 2.31 µmol/(g min) | 0% | 5% | 30% | 0% | 5% | 30% |  | 23% |
| **Simulation results** |  |  |  |  |  |  |  |  |
| Fitted CMRO_2_ in µmol/(g min), as mean (std) | 2.41 (0.61) | 2.52 (0.71) | 3.08 (0.50) | 3.12 (2.81) | 3.32 (2.86) | 3.53 (2.19) |  | 3.42 (2.17) |
| Cohen’s d |  | 0.16 | 1.18 |  | 0.07 | 0.16 |  | 0.12 |
| Calculated sample size |  | 243 | 7 |  | 1264 | 243 |  | 431 |
| Maximum in mmol/L, as mean (std) | 24.9 (0.39) | 25.2 (0.39) | 26.4 (0.39) | 24.9 (0.78) | 25.1 (0.80) | 26.4 (0.76) |  | 26.1 (0.81) |
| Cohen’s d |  | 0.59 | 3.89 |  | 0.32 | 1.99 |  | 1.51 |
| Calculated sample size |  | 20 | 3 |  | 62 | 4 |  | 5 |

# Supplementary Figures

**
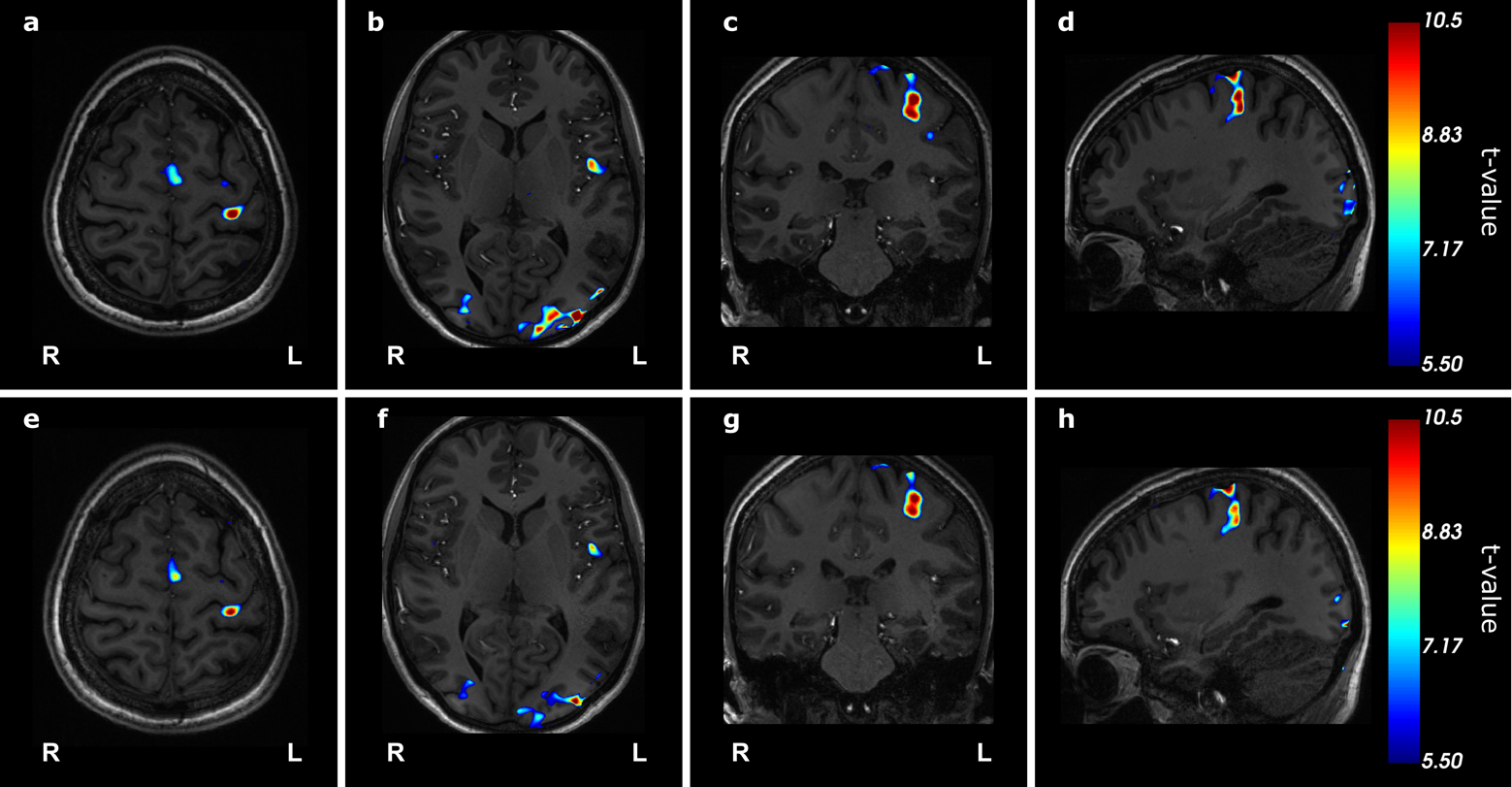
**

**Figure S1: MPRAGE and BOLD images of P5 in a)/e) axial view (higher slice), b)/f) axial view (lower slice) c)/g) coronal view, and d)/h) sagittal view at timepoint t = 0 min for a)-d) and t = 40 min for e)-h).** After 40 min of continuous sensorimotor and visual stimulation, the significant BOLD activity remains about the same in the sensorimotor cortex and is slightly decreased in the visual cortex.


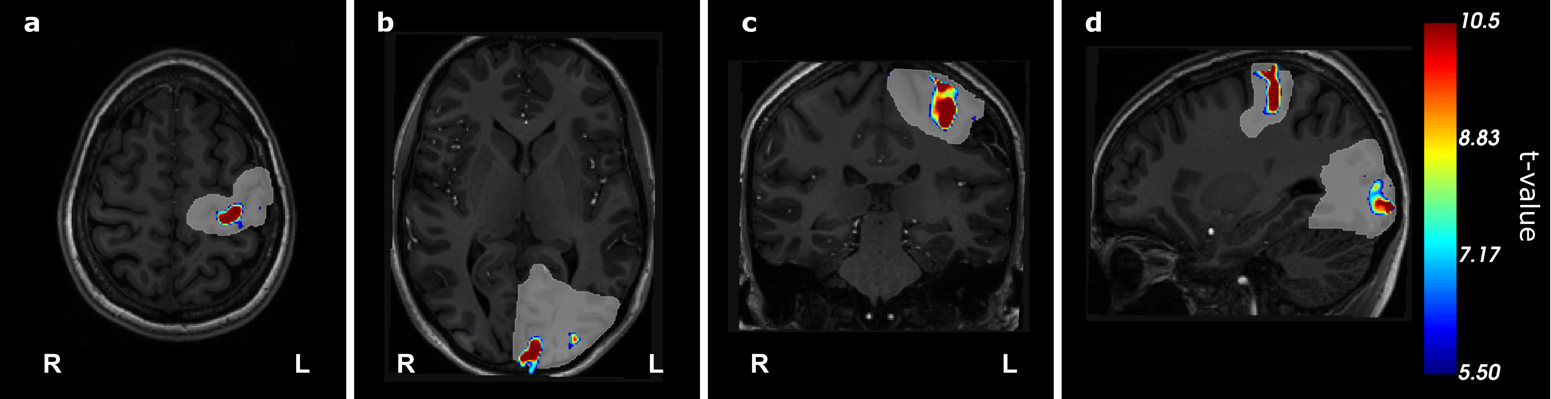


**Figure S2: MPRAGE and BOLD images of P5 in a) axial view (higher slice), b) axial view (lower slice) c) coronal view, and d) sagittal view.** The white ROI shows the left sensorimotor and visual cortex used for data analysis of the dynamic ^17^O MRI data set. Overlaid is the BOLD activity map, showing significant activity in the chosen ROI.

**
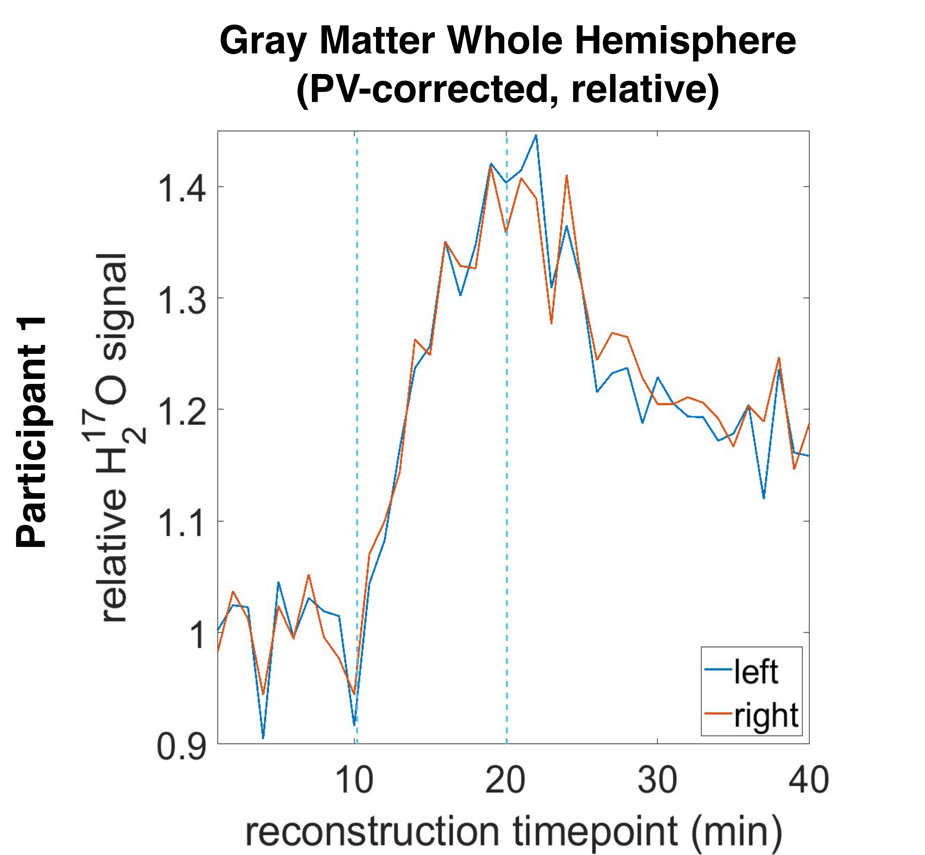
**

**Figure S3: Relative PV-corrected data of P1 of the complete GM of the left (blue) vs. right (orange) hemisphere.** Even for these larger ROI the signal evolution shows a high noise level.

**References**

1. Ebersberger L, Kratzer FJ, Potreck A, Niesporek SC, Keymling M, Nagel AM, Bendszus M, Wick W, Ladd ME, Schlemmer H-P, Hoffmann A, Platt T, Paech D (2023) First application of dynamic oxygen-17 magnetic resonance imaging at 7 Tesla in a patient with early subacute stroke. Frontiers in Neuroscience 17.

2. Atkinson IC, Thulborn KR (2010) Feasibility of mapping the tissue mass corrected bioscale of cerebral metabolic rate of oxygen consumption using 17-oxygen and 23-sodium MR imaging in a human brain at 9.4 T. Neuroimage 51 (2):723-733.

3. Niesporek SC, Umathum R, Lommen JM, Behl NGR, Paech D, Bachert P, Ladd ME, Nagel AM (2018) Reproducibility of CMRO2 determination using dynamic (17) O MRI. Magn Reson Med 79 (6):2923-2934.

4. Cohen J (1988) Statistical Power Analysis for the Behavioral Sciences. L. Erlbaum Associates,

5. Sawilowsky SS (2009) New effect size rules of thumb. Journal of Modern Applied Statistical Methods 8 (2):597-599.
